# The role of autophagy induced by tumor microenvironment in different cells and stages of cancer

**DOI:** 10.1186/s13578-015-0005-2

**Published:** 2015-03-28

**Authors:** Xue Yang, Dan-Dan Yu, Fei Yan, Ying-Ying Jing, Zhi-Peng Han, Kai Sun, Lei Liang, Jing Hou, Li-Xin Wei

**Affiliations:** Tumor Immunology and Gene Therapy Center, Eastern Hepatobiliary Surgery Hospital, The Second Military Medical University, 225 Changhai Road, 200438 Shanghai, China; Central laboratory, Ren Ji Hospital, School of Medicine, Shanghai JiaoTong University, Shanghai, China; Department of Pharmacy, Chang Hai Hospital, The Second Military Medical University, Shanghai, China

**Keywords:** Autophagy, Tumor microenvironment, Tumorigenesis

## Abstract

Development of a tumor is a very complex process, and invasion and metastasis of malignant tumors are hallmarks and are difficult problems to overcome. The tumor microenvironment plays an important role in controlling tumor fate and autophagy induced by the tumor microenvironment is attracting more and more attention. Autophagy can be induced by several stressors in the tumor microenvironment and autophagy modifies the tumor microenvironment, too. Autophagy has dual roles in tumor growth. In this review, we discussed the interaction between autophagy and the tumor microenvironment and the paradoxical roles of autophagy on tumor growth at different stages of tumor development.

## Introduction

Cancer cells are surrounded by a complex milieu. This cancer cell niche is called the tumor microenvironment, and it contributes to the development and metastasis of tumors. The tumor microenvironment is a new emerging concept in tumor research and has become a research hallmark. The tumor microenvironment not only contributes to cancer cell survival by supplying nutrients but also contributes to tumor cell invasion and metastasis. Thus, detecting the composition and function of the tumor microenvironment is important for understanding the mechanisms of tumorigenesis and tumor metastasis to improve therapeutic strategies. The tumor microenvironment is a complex system and is difficult to study. It is composed of various stromal cells, including fibroblasts, vascular endothelial cells, immune cells, adipocytes, mesenchymal stems cells (MSCs), and various cytokines [1]. The physiological characteristics of the tumor microenvironment are clearly different from those of normal tissues and have been described as hypoxic, nutrient deprived, energy limited, acidic, and inflammatory [2-8]. These characteristics can induce autophagy by activating various pathways [5-8]; thus, autophagy can shape the tumor microenvironment. The crosstalk between autophagy and the tumor microenvironment is attracting increasing attention, as the tumor microenvironment shaped by autophagy may play a crucial role in modulating tumor development, metastasis, and therapeutic resistance.

## Tumor microenvironment-induced autophagy

**Figure 1:**
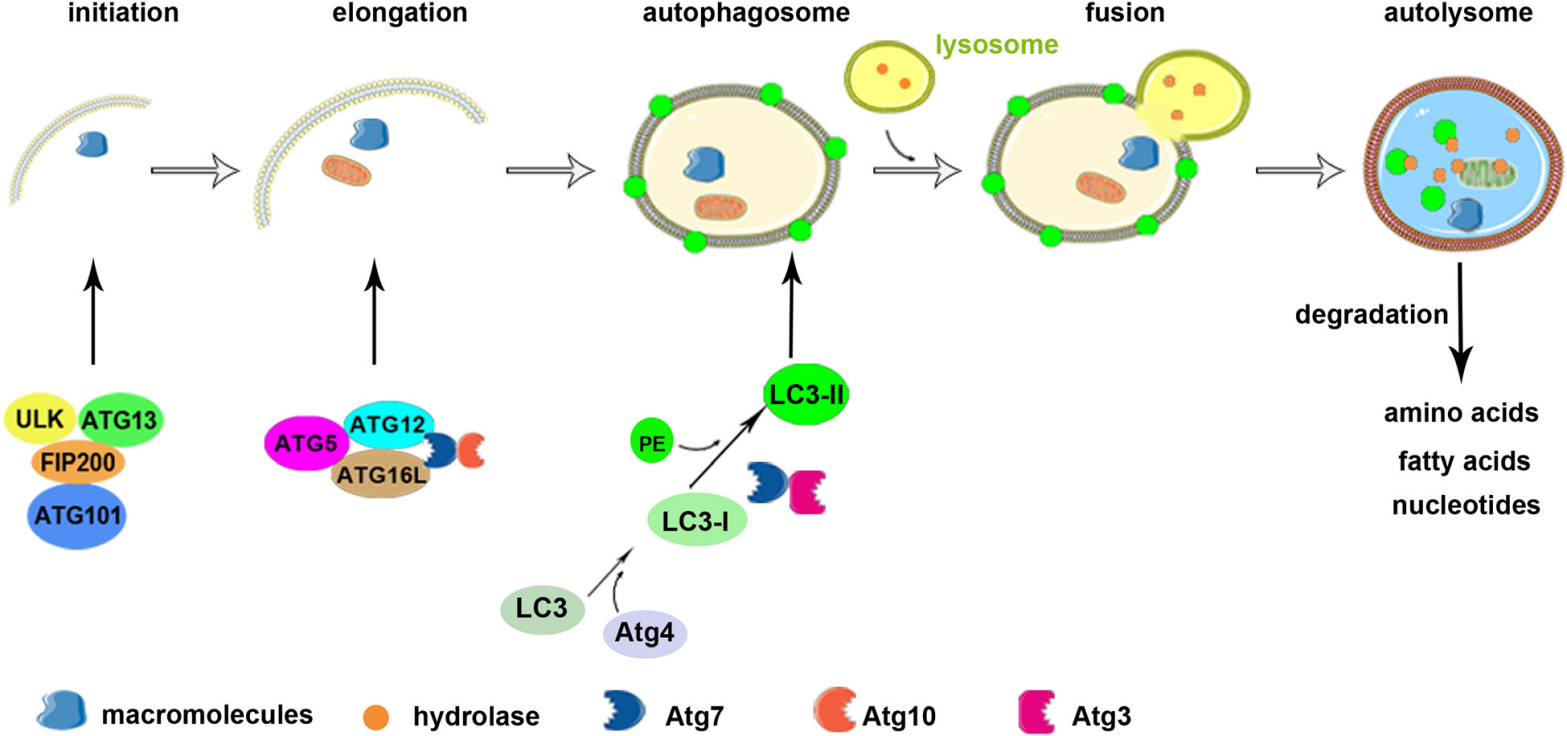
**Process of autophagy. Autophagy includes five phases: initiation, elongation and autophagosome formation, fusion, and autolysosome formation.** Macromolecules are targeted to double-membrane vesicles called autophagosomes and then autolysosomes form by fusion with lysosomes. Autophagy is initiated by the ULK1 complex containing ULK, Atg13, FIP200, and Atg101. Autophagosome elongation and maturation involves two ubiquitin-like conjugation systems, such as the microtubule-associated protein 1 light chain 3 (LC3) and the Atg12 systems. The autophagosome fuses with a lysosome to form an autolysosome, which degrades macromolecules into amino acids, fatty acids, and nucleotides.

Autophagy is an evolutionarily conserved catabolic pathway from yeast to mammals that serves as a major lysosomal degradation pathway for recycling intracellular components. Autophagy is emerging as the key process that eliminates damaged macromolecules, including proteins, lipids, and dysfunctional organelles. Autophagy is a complex process comprising many steps (Figure 1), including initiation, elongation, and autophagosome and autolysosome formation. Macromolecules are targeted to double-membrane vesicles called autophagosomes, and autolysosomes form by fusion with lysosomes [9]. Cytoplasmic constituents are degraded and digested by lysosomal enzymes in the autolysosome for recycling and reuse. The products of degradation, such as amino acids, fatty acids, and nucleotides, are essential for cell growth. The multi-step autophagic process is regulated by a limited number of highly conserved genes known as autophagy-related genes (ATGs) [10].

The formation of autophagosomes is initiated in mammalian cells primarily by the Unc51-like kinase 1 (ULK1) complex containing ULK, Atg13, FIP200, and Atg101 [11-13]. Activation of this complex can be inhibited by mammalian target of rapamycin (mTOR) complex 1, which is a master negative regulator of autophagy in several pathways [14,15]. Elongation and maturation of autophagosomes involves two ubiquitin-like conjugation systems, such as the microtubule-associated protein 1 light chain 3 (LC3) system and the Atg12 system [16]. Atg12 is conjugated to Atg5 by Atg7 (E1 enzyme) and Atg10 (E2 enzyme). The Atg12-Atg5 heterodimer interacts with Atg16L, and this complex promotes elongation of the autophagic membrane [17]. LC3I is formed immediately by Atg4B cleaving a free glycine residue after the full-length LC3 precursor is translated. After autophagy is induced, phosphatidylethanolamine (PE) is conjugated with LC3I (called LC3II) by Atg7 (E1 enzyme) and Atg3 (E2 enzyme). PE-conjugated LC3 becomes an insoluble form (LC3-II) that is stably inserted into the autophagosomal membrane [18].

**Figure 2:**
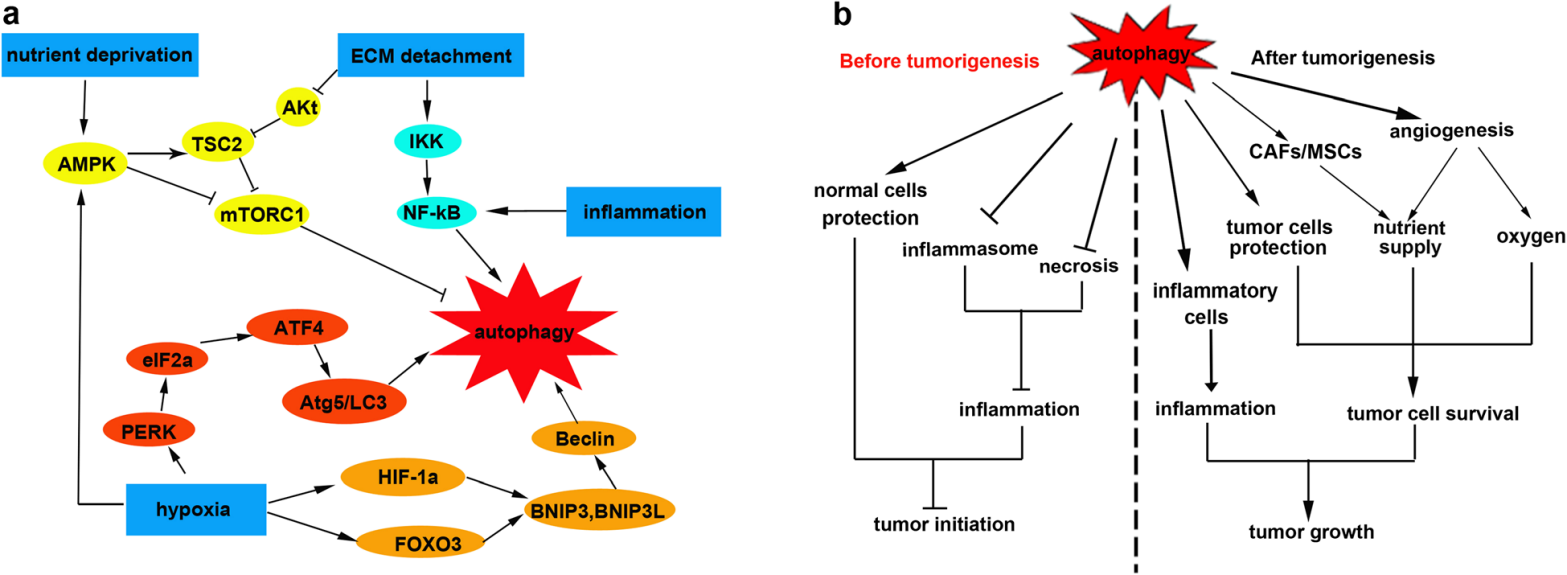
**Paradoxical roles of autophagy induced by the tumor microenvironment during different stages of tumor development. (a)** Autophagy is induced by multiple stressors in the tumor microenvironment, including hypoxia, nutrient deprivation, inflammation, and ECM detachment. Hypoxia activates autophagy through AMPK, PERK and HIF-1a/FOXO3 signaling pathways. The AMPK pathway is also activated by nutrient deprivation. Autophagy can be also induced by ECM detachment through the Akt-mTORC1 and IKK pathways. Inflammation is another inducer of autophagy that acts through the NF-κB signal pathway. **(b)** Before tumorigenesis, autophagy can suppress tumor initiation by protecting normal cells and inhibiting inflammation (including inhibiting inflammasome and necrosis). In contrast, autophagy promotes tumor growth in established tumors. Autophagy can promote inflammation by favouring inflammatory cells growth. Autophagy can also protect tumor cells and provide sufficient oxygen and nutrient.

Autophagy occurs at a basal level in every cell as housekeeping and plays key roles in cell development, immunity, tissue remodeling, and orientation with the surrounding environment. In addition, in the tumor microenvironment, autophagy is also activated in response to multiple metabolic stressors (Figure 2a), such as oxygen/nutrient deprivation, and degradation of the extracellular matrix (ECM) [19,20].

Autophagy benefits cells suffering in an unfavorable microenvironment by eliminating garbage and preventing accumulation of toxins. In contrast, autophagy also supplies energy and compounds for cell survival and metabolism. Thus, autophagy serves as an inherently cytoprotective mechanism by self-eating [13].

### Hypoxia and anoxia

The vasculature is insufficient to supply adequate oxygen when solid tumor diameter is > 2 mm, resulting in local hypoxic and anoxic conditions (oxygen concentrations < 3% and < 0.1%, respectively) inside the tumor [21]. Increasing evidence indicates that 50–60% of tumors grow under hypoxic conditions [22-25], and that enhanced autophagy promotes tumor cell survival [2]. Hypoxia within the immediate area of a tumor arises mainly because the balance between oxygen consumption and supply is disrupted. The supply of oxygen to normal tissues and organs meets their metabolic requirements, whereas oxygen consumption may outweigh the insufficient supply to tumors, resulting in locally low oxygen levels in tumor tissues. Hypoxia occurs in tumor tissues for the following reasons: (1) abnormal microvessel structure and function, which is known as perfusion-limited oxygen delivery, and exists transiently; (2) limited oxygen diffusion due to increased transport distance (>70 μm), which is called diffusion-limited oxygen delivery; and (3) impaired blood oxygen-carrying capacity induced by tumor-associated or therapeutic-induced anemia, which is termed anemic hypoxia [26].

Hypoxia-induced autophagy mainly depends on hypoxia-inducible factors (HIFs), whereas anoxia-induced autophagy is HIF independent [27,28]. HIFs are a family of heterodimers containing a constitutive subunit and an oxygen-regulated subunit that are only expressed when oxygen concentration declines below a 5% threshold. HIF-1α activates transcription of BNIP3 and BNIP3L (BNP3-like protein, also known as NIX) under moderate hypoxia, which disrupts the Beclin 1/Bcl-2 complex, releasing Beclin1 and activating autophagy [28-31], as Beclin1 is an autophagy-activator gene. Moreover, transcription of BNIP3 and BNIP3L is upregulated by the FOXO3 transcription factor. Mammucar et al. showed that FOXO3 overexpression upregulates LC3 expression and increases LC3 lipidation in skeletal muscle cells, resulting in activation of autophagy [32]. FOXO3 also plays a key role in activating hematopoietic stem cells under nutrient-deprived conditions [33]. BNIP3L, which is often present on the outer mitochondrial membrane, modulates elimination of mitochondria by autophagy (mitophagy). HIF-2 also regulates autophagy in chondrocytes and surprisingly inhibits HIF-1α function [34]. Other pathways involved in hypoxia-induced autophagy include the protein DJ-1 pathway (also called CAP1/RS/PARK7), the platelet-derived growth factor receptor-dependent pathway, and the unfolded protein response (always elicited by endoplasmic reticulum stress) [28,35-37]. Furthermore, inhibiting mTOR and stimulating of 5′ AMP-activated protein kinase (AMPK) contribute to inducing autophagy. Hypoxia-induced autophagy also requires eIF2α phosphorylation mediated by PERK to reach the maximum level [38]. Hypoxia increases transcription of the essential autophagy genes LC3 and Atg5 by activating the transcription factors ATF4 and CHOP, respectively, both of which are regulated by PERK [38].

### Nutrient deprivation

Proliferating cancer cells must sustain intracellular energy and nutrient levels to survive, but essential ingredients in the microenvironment at the early stage of tumorigenesis are insufficient for cancer cell survival [39]. Thus, cancer cells are nutrient deprived and to survive must cope with this stress using available metabolic pathways. Nutrient (including amino acids and glucose) depletion is the most potent physiological inducer of autophagy. Several studies have shown that autophagy plays a critical role in protecting cells against nutrient depletion [40,41]. Ammonia, generated from deamination of glutamine in mitochondria, stimulates autophagic flux in an autocrine and/or paracrine manner [42]. Amino acids, particularly branched-chain amino acids, activate mTORC1 and inhibit autophagy. Thus, the absence of amino acids induces autophagy by regulating mTOR activity [43]. In addition to amino acids, cells require sufficient ATP supplied by glucose, so a lack of glucose will activate autophagy to sustain energy homeostasis [44]. In addition, glucose deprivation may induce autophagy by oxidative stress [45]. Moreover, receptor for advanced glycation end product (RAGE) activates autophagy by inhibiting mTOR and blocks apoptosis in pancreatic cancer cells, which increases their survival [46]. Another report demonstrated that autophagosomes form in colorectal cancer cells under amino acid- and glucose-deprived conditions, which may contribute to survival of the cancer cells [39].

Autophagy can also be stimulated by activating the AMPK pathway to protect cancer cells against nutrient starvation. AMPK holds the core node that integrates several autophagy-inducing stimuli. The AMP: ATP ratio is a key factor for AMPK to monitor energy. Several upstream AMPK kinases, including liver kinase B1 (activated by energy depletion), calcium/calmodulin kinase kinase-ß (activated by cytosolic Ca^2+^), and transforming growth factor (TGF)-ß-activated kinase-1 (involved in activating IKK) activate AMPK by phosphorylating a threonine residue on its catalytic α-subunit [47]. The best-studied mechanisms by which AMPK induces autophagy are inhibiting mTORC1, phosphorylating tuberous sclerosis complex 2, and regulating the mTOR-associated protein, Raptor. mTOR can also be inhibited by a growth factor deficiency, such as insulin and insulin-like growth factor [21].

### Inflammation

Inflammation is a cellular response that occurs at cell and tissue injury sites. Tumors were described as unhealed wounds by Dvorak over 20 years ago and were believed to produce inflammatory mediators, such as cytokines and chemokines, continuously. Tumor progression is accompanied by increased expression of inflammation-associated genes [48]. It has been widely accepted that chronic inflammation promotes cell malignancy and tumorigenesis. Bulk inflammatory cytokines, such as tumor necrosis factor (TNF)-α, interleukin (IL)-6, TGF-β, and IL-10, contribute to the occurrence and development of cancer. In 1863, Rudolf Virchow proposed that inflammation plays a key role in tumor progression. He found that inflammatory cells infiltrate tumors [49]. His hypothesis was proved by recent studies that infection and chronic inflammation-associated disease can drive cancers [50].

The initial goal of inflammation is to eradicate foreign bodies and tumors [51]. Tumor tissues not only stimulate an adaptive immune response by recruiting T lymphocytes, dendritic cells (DC), and occasional B cells but also mediate the innate immune response by recruiting macrophages, polymorphonuclear leucocytes, and rare natural killer cells. However, if the inflammation cannot be controlled, surrounding tissues may be subjected to malignant transformation. Cytokines in the tumor microenvironment regulate the anti-tumor response, but cytokines induce cancer in another way during chronic inflammation [52]. Inflammation increases the risk of tumorigenesis due to the bioactive molecules secreted by cells that have infiltrated the tumor milieu, including cytokines, growth factors, and chemokines that maintain cell growth, limit apoptosis, and promote angiogenesis [53]. Furthermore, inflammation can activate autophagy [54].

Tumor cells induce oxidative stress in adjacent fibroblasts; thus, inducing nuclear factor kappa B (NF-κB) and activating HIF-1α, which stimulate autophagy [55-57]. Furthermore, NF-κB is a key regulator of the inflammatory response, suggesting a close connection between inflammation and autophagy. Ubaldo et al. showed that co-culturing fibroblasts with tumor cells activates the NF-κB signaling pathway and induces a cytokine storm that includes IL-6, IL-8, IL-10, macrophage inflammatory protein 1-α, interferon-γ, RANTES, and granulocyte-macrophage colony-stimulating factor. Treatment with these cytokines can drive autophagy [58]. Thus, it has been speculated that inflammation plays a positive role in the occurrence of autophagy.

### Detachment of the ECM

The attachment of epithelial cells to the ECM is mediated by integrin and is vital for cell growth and survival [59]. Loss of ECM attachment leads to a type of apoptosis known as anoikis [60]. Some studies have shown that a lack of appropriate ECM contact also robustly induces autophagy to promote cell survival, either during early formation of carcinoma or in the later stages of dissemination and metastasis [61,62]. Moreover, components of the ECM regulate autophagy and mitigate its role in cell survival, and adhesion of HeLa cells to collagen I or IV is one of the mechanisms [63]. Another study of MCF10A mammary epithelial cells (MECs) in a three-dimensional culture system showed that autophagy is rapidly induced to enhance cell survival during anoikis when cells are grown under low ECM attachment conditions [64]. Depletion of human ATGs, such as ATG5, ATG6 and ATG7, by si-RNA inhibits matrix detachment-induced autophagy [65]. Chen et al. reported that the phosphatidylinositol-3-kinase (PI3K)-AKT-mTORC1 pathway is a major regulator of autophagy in detached mouse fibroblasts. Activation of the PI3K-AKT-mTORC1 pathway decreases during ECM detachment, which is correlated with the activation of autophagy. Activation of the IKK complex plays a key role promoting autophagy in MECs deprived of ECM contact [66]. Thus, both the PI3K-AKT-mTORC1 and IKK pathways are important regulators of autophagy during ECM detachment.

## Autophagy shapes the tumor microenvironment

As described before, multiple stressors drive autophagy in the tumor microenvironment, and increasing evidence shows that autophagy modifies the tumor microenvironment through different pathways. [13]. Autophagy is a key lysosomal pathway that degrades macromolecules, including intracellular organelles, denatured proteins, and nucleic acids and contributes to cell reconstruction, regeneration, and repair by recycling and reusing cellular contents. In the tumor microenvironment, autophagy may display different properties.

### Autophagy promotes angiogenesis

Autophagy has important roles in tumor angiogenesis. Autophagy-associated genes in endothelial cells, such as Atg5, modulate starvation and hypoxia-evoked angiogenesis, which may occur through the high mobility group box 1 (HMGB1) pathway [67]. HMGB1 is a major chromatin-associated protein that translocates to the cytoplasm and is released from endothelial cells under stress [68]. HMGB 1 is released by damaged or dead cells and acts as an inflammatory cytokine and damage-related protein. In addition, extracellular HMGB 1 elicits autophagy by binding to Beclin 1 [69]. HMGB 1 is an important effector of the crosstalk between endothelial cells and tumor cells and favors angiogenesis and tumor cell survival in a hypoxic microenvironment.

### Autophagy supplies nutrients

Normal fibroblasts adjacent to tumor cells undergo reprogramming during tumor development by interacting with tumor cells. Fibroblasts acquire the myofibroblast phenotype and are known as cancer-associated fibroblasts (CAFs). Lisanti et al. revealed that heightened autophagy in CAFs plays a key role in energy support for neighboring epithelial tumor cells and favors their survival [70]. When CAFs overexpressing pro-autophagic molecules were co-injected with cancer cells into immunocompromised mice, they promoted tumor growth and lung metastasis. In contrast, activating autophagy in cancer cells reduces tumor growth [71]. This result suggests that CAFs supply sufficient energy for tumor cell growth by generating a fertile stroma and nutrients and that autophagy plays different roles in various compartments [72]. However, it remains unclear how the paradoxical roles of autophagy in tumors are regulated. Increasing evidence indicates that the role of autophagy in tumors is far more complex than thought previously [13].

Suppressing autophagy in apoptosis-deficient tumor cells impairs their survival under stress conditions *in vivo* and *in vitro*, indicating that autophagy sustains cell survival when nutrients are limited [2]. Cells undergoing autophagy provide nutrition by degrading intracellular proteins and organelles. Autophagy ensures metabolism of hematopoietic stem cells during trophic factor deprivation [73]. Moreover, autophagy sustains nutrient metabolism when nutrient levels are low during mouse development [74]. All of these results show that autophagy favors cell metabolism and energy balance. In contrast, defects in autophagy increase the incidence of many cancers, such as human breast, ovarian, and prostate cancers [75-77].

### Autophagy regulates the inflammatory response

Growing evidence in the last 10 years suggests that inflammation plays a key role in tumor occurrence and development. Autophagy has been also proposed to be a key regulator of inflammation through various mechanisms, as an autophagy deficiency increases necrosis and inflammation in tumor cells, whereas activating autophagy has the opposite effect. White’s team demonstrated in 2006 that impaired apoptosis and autophagy induces necrosis; thus, stimulating the inflammatory response and accelerating tumor growth [21]. All of these results indicate that autophagy plays a key role in cell death and inflammation induced by necrosis.

Unlike cell apoptosis, necrosis provokes an inflammatory storm. HMGB1 released from necrotic cells activates NF-κB after binding to the cell surface receptor RAGE [78,79]. Nucleic acids from necrotic cells elicit inflammation through a Toll-like receptor. Several studies have shown that autophagy blocks two forms of necrotic cell death, such as necroptosis and poly-ADP-ribose polymerase (PARP)-mediated cell death. Necroptosis is dependent on caspase and induced by a cell death ligand, such as TNF-α and FasL. PARP-mediated cell death is another form of programmed-necrotic cell death that can be induced by DNA damage [80,81]. In contrast, inhibiting the autophagy-related genes Atg5 and Beclin 1 results in increased sensitivity of cells to necrotic death [82] IL-1α released from necrotic cells activates Kupffer cells, which produce cytokines, induce TNF-α, IL-6, and hepatocyte growth factor and activate NF-κB, leading to hepatocarcinogenesis [83]. Massey et al. showed that a deficiency in Atg16L1 is involved in Crohn’s disease, suggesting a potential role of autophagy in promoting inflammation [84]. Autophagy regulates inflammatory signals directly. Inflammasomes can activate under autophagy-deficient conditions and promote maturation of inflammatory cytokines including IL-1β and IL-18 [85]. Further studies showed that mitochondrial reactive oxygen species (mtROS) produced by damaged mitochondria play a crucial role in this process. ROS activate the NLRP3 inflammasome, which promotes caspase 1 maturation. Activated caspase 1 cleaves pro-IL-1β to produce matures IL-1β that is subsequently secreted by cells [86]. In addition, mtROS also act as signaling molecules to trigger other inflammatory cytokines, such as TNF-α and IL-6 [87]. Furthermore, autophagy affects immune cells directly. Neutrophils, as the first immune cells migrating to a tumor inflammatory site, promote inflammation and activate macrophages and DCs [88]. The activation of autophagy in neutrophils mediates death of neutrophils, which results in decreased inflammation [89]. In contrast, antigen-presenting cells (APCs), such as macrophages and DCs, undergo autophagy to survive under stress conditions [90].

Autophagy stimulates the innate and adaptive immune responses. Autophagy is involved in activating DCs [91,92]. Autophagy in T cells has also been investigated. Autophagy is activated in both CD4+ and CD8+ T cells to promote their proliferation [93,94]. CD4+ and CD8+ cells deficient in ATG3, ATG5 and ATG7 cannot proliferate after they are activated [95,96], and Atg5−/− lymphocytes fail to repopulate the periphery due to overwhelming cell death [93]. Autophagy in APCs and T cells promotes the inflammatory response.

## Paradoxical roles of autophagy during different stages of tumor development

Tumor microenvironment-driven autophagy have different roles in different stages of tumor development [2,3,97] (Figure 2b), the mechanism of which remains unclear. There has been work suggesting that autophagy is activated in different cells during different stages of tumor development, thus results in different effects on tumor growth.

### Autophagy inhibits early tumorigenesis

Autophagy was initially considered to be a process that suppressed malignant transformation. The first direct evidence of the relationship between autophagy and cancer was established in 1999, when Levine et al. discovered that Beclin1 is a candidate tumor suppressor gene [77]. They found that Beclin1 is monoallelically deleted in a high percentage of human breast and ovarian cancers, and that Beclin1 expression is frequently low in human breast cancer, including cell lines and cancer tissues. Moreover, increased Beclin1 expression in breast cancer cell lines inhibits cell proliferation *in vitro* and tumor generation in nude mice [77]. Then, the importance of single copy loss of the Beclin1 gene was exhibited in Beclin1 heterozygous knockout mice, which are prone to develop spontaneous lymphomas, lung cancers, and liver cancers, as well as accelerated hepatitis B virus-induced hepatocarcinogenesis [75]. Levine et al. further found that Akt suppresses autophagy by mTOR-independent phosphorylation of Beclin1 and ultimately promotes tumorigenesis [98].

Many other autophagy machinery components besides Beclin1 play tumor-suppressive roles in tumorigenesis. Atg4C knockout mice have increased susceptibility to develop carcinogen-induced fibrosarcomas [99]. UV radiation resistance associated gene and Bif-1, which are components of the Beclin1/class III PI3K complex, also participate in controlling cell proliferation and suppressing tumorigenesis [100,101]. Notably, Atg5 mosaic-deleted mice develop spontaneous benign liver tumors, but no tumors are detected in other organs. Liver-specific deletion of Atg7 also leads to benign liver tumors in a mice model [102]. Atg5 −/− or Atg7−/− mice or mice hypomorphic for Atg16L1 exhibit intestinal Paneth cell abnormalities resembling Crohn’s disease, which may results in intestine cancer [103,104]. Atg4, Atg 5, Atg 12 and Atg 9b have also been demonstrated to be deleted or mutated in various human cancers [7]. These reports suggest that tumor suppression may be a property of the autophagy machinery but is not associated with a signal autophagy protein. Tumors in both studies were benign hepatic adenomas but not frank cancer, suggesting that loss of autophagy may be a trigger for primary tumorigenesis, but not for malignant progression during late tumorigenesis.

### Autophagy promotes established tumor growth

In addition to tumor suppression role of autophagy in the initial process of tumorigenesis, autophagy seemingly plays an opposite role as a tumor promoter in established cancers. Several studies have shown that autophagy promotes survival of tumor cells under several stressors [21]. Degenhardt et al. showed that activation of autophagy in evolving tumors promotes tumor survival [2]. Another study by Sun suggested that autophagy suppresses hepatocarcinogenesis during the dysplastic stage and promotes hepatocarcinogenesis at the tumor-forming stage [105]. Except for the difference in tumor type, this strange phenomenon may result from differences in the incipient cells involved with tumor development. Altman et al. found that a deficiency of autophagy following deletion of Atg3 aggravates BCR-Abl-expressing hematopoietic precursor cell death under stress and prevents BCR-Abl-mediated leukemogenesis [106]. White’s group also found that Ras expression upregulates basal autophagy, which was required for survival of immortal mouse kidney epithelial cells during starvation and during Ras-mediated tumorigenesis [107]. A study in a conditional FIP200-knockout mouse model showed that inhibiting autophagy retards MMTV-PyMT-mediated tumorigenesis of mammary epithelial cells by impairing tumor cell survival and proliferation [108].

The process of tumorigenesis involves activation of various oncogenes and inactivation of anti-oncogenes. Autophagy may mainly impact tumor cells and consequently play a role as a tumor promoter during oncogene-mediated tumor development. However, it appears that the protumorigenic role of autophagy extends beyond the hypoxia/nutrient deprived regions of a tumor. Detachment of the ECM during early carcinoma formation or in the later stages of dissemination and metastasis robustly induces autophagy to promote cell survival. Autophagy also mediates therapeutic resistance in a variety of situations [97]. Tumor cells can survive after chemo- or irradiation therapy by activating autophagy. Li et al. showed that inhibiting autophagy with 3-methyladenine or by targeting Atg7 enhances the 5-fluorouracil treatment effect in human colorectal cancer cells [109]. Inhibiting autophagy also enhances the therapeutic efficacy of cisplatin and 5-fluorouracil in esophageal and colon cancer cells, respectively [109,110]. The autophagy inhibitor chloroquine (CQ) and hydroxychloroquine (HCQ) have been used to enhance the anti-tumor effects of toxic drugs in clinical trials. Furthermore, autophagy may be a key modulator of tumor metastasis [111] by regulating the epithelial-mesenchymal transition, which can be induced in the tumor microenvironment.

### Autophagy has different roles in different cells

#### Normal cells

Normal cells face the unfavourable microenvironment and are involved in metabolic stress in early stage of tumorigenesis. Metabolic stressors distinctly impact on cellular genome stability. Metabolic stressors disturb the mechanisms of DNA synthesis/repair by accumulating misfolded and aggregate-prone proteins, and reactive oxygen species (ROS)-generating organelles, especially mitochondria. In autophagy-competent cells, autophagy clears these accumulations to limit the metabolic stress. In contrast, autophagy defection sensitizes normal cells to metabolic stress, and results in DNA damage increase, gene amplification and aneuploidy increase, and ultimately promotes tumorigenesis [3,112]. These data showed that autophagy plays an important role in the protection of genomic stability in normal cells and inhibiting tumorigenesis. In addition, autophagy deficiency leads to differentiation disorder and abnormal proliferation of stem cells, both of which may be the early events in the process of tumorigenesis [113,114].

#### Cancer cells

It has been known that autophagy not only benefits normal cells suffering from metabolic stress, but also protects cancer cells in the stress microenvironment during tumor development. In the rapidly growing stage of tumor development, angiogenesis can not satisfy the great demand of amino acids, oxygen and growth factors for fast-proliferating tumor cells. Autophagy can digest damaged proteins, organelles and other macromolecules and recycle cytoplasmic materials to balance the demand of nutrients and energy [115]. And a series of metabolic stressors (including starvation, hypoxia, and ROS accumulation) induce autophagy for survival in tumor cells [116]. In addition to metabolic stress-activated autophagy, autonomous autophagy also plays a crucial role in tumor development. Recently, many studies have shown that several types of tumor cells require autonomous autophagy for tumor growth in normal condition [107,117].

#### Tumor-associated mesenchymal stem cells

In tumors, there are various cell types constituting the tumor stroma [13]. Mesenchymal stem cells is an important component of tumor stroma and have multiple roles in the tumor microenvironment [118]. The effect of MSCs on tumor cells in stressful conditions has also been described. MSCs promote survival and drug resistance in various hematological tumors [119-121]. Recent studies have established that MSCs provide sufficient stromal support for tumor cells [122]. The effect of MSCs on tumor growth in the tumor microenvironment may be related to activating autophagy. Cecilia *et al*. demonstrated that serum-deprived MSCs (SD-MSCs) express Beclin1, ATG10, and ATG12 and the SD-MSCs undergoing autophagy provide the needed nutrients and secrete survival and anti-apoptotic factors for self-survival and survival of surrounding cells [123].

## Conclusion

Tumor microenvironment is very important to tumor growth, metastasis and therapy resistance and attracts more and more attention. Studies have shown that autophagy is an important factor in the tumor microenvironment. The mechanism of autophagy interfaced with tumor microenvironment still remains unclear and needs to be explored. But previous studies suggested that autophagy is not only a result of the tumor microenvironment, but also has effect on it. The tumor microenvironment can activate autophagy through different pathways and autophagy can modify the tumor microenvironment by promoting angiogenesis, supplying nutrients, and modulating the inflammatory response and thus help the cells in the tumor microenvironment overcome metabolic stress, maintain homeostasis, and survive in poor microenvironment.

**Figure 3:**
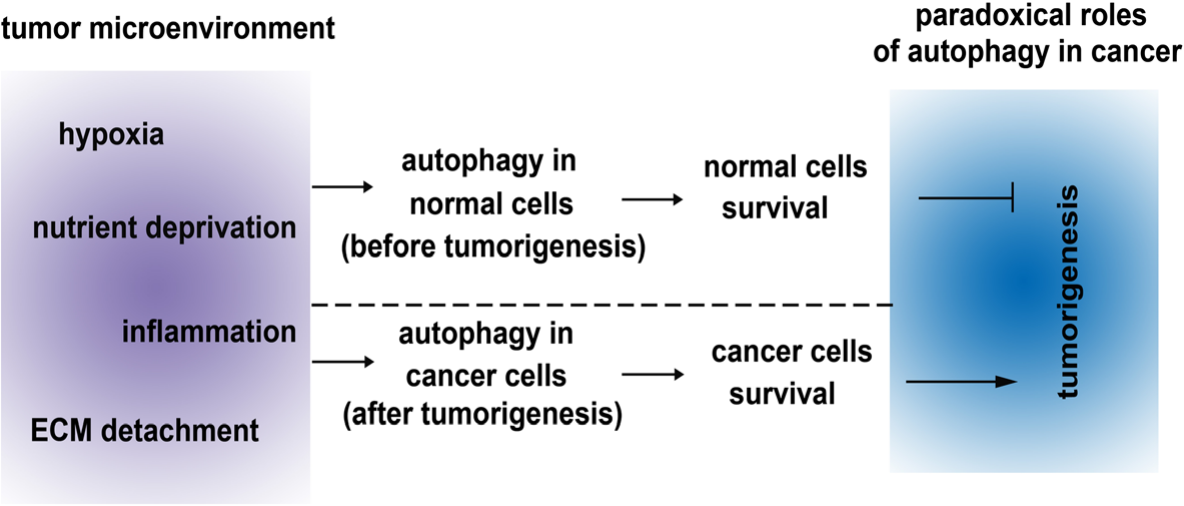
**Concise summary of relation between autophagy and tumor growth.** Autophagy can be induced by different metabolic stressors in the tumor microenvironment including hypoxia, nutrient deprivation, inflammation and ECM detachment. Autophagy can be activated in different cells at different stages of tumor growth and have paradoxical roles in tumor growth. Before tumorigenesis, autophagy promotes normal cells survival and suppresses tumorigenesis. In contrast, after tumorigenesis, autophagy promotes survival of the cancer cells and thus promotes tumor growth.

Tumor resistance to radiotherapy and chemotherapy is also associated with autophagy. Various tumor-associated animal models have confirmed that autophagy inhibition has the effects of enhancing chemosensitivity and promoting tumor regression. Of the known autophagy inhibitors, only CQ/HCQ have been evaluated in human clinical trials [124].There have been more than 50 clinical trials working on CQ/HCQ efficacy in various human cancers, particularly in advanced or metastatic cancers (https://clinicaltrials.gov). A combination of an autophagy inhibitor with a toxic drug possibly is an alternative treatment for advanced or metastatic cancer, and HCQ may be preferred to CQ due to its more acceptable side effects [125]. However, autophagy-targeted therapy still should be cautious because autophagy has dual roles in tumor growth progress (Figure 3). The dual roles of autophagy in tumor growth may be tissue-dependent and vary among different stages of tumor growth. Thus, autophagy inhibitor may play different roles in different stages and types of tumor growth. In addition, excess and sustained autophagy leads to tumor cell death or senescence, which results in tumor dormancy. Therefore, using autophagy as a tumor treatment target should be further investigated and studying autophagy in tumor microenvironment is very important for discovery of new therapeutic targets.

## Abbreviations

ATG: Autophagy-related genes
mTOR: Mammalian target of rapamycin complex
LC3: Light chain 3
PE: Phosphatidylethanolamine
ECM: Extracellular matrix
HIFs: Hypoxia-inducible factors
RAGE: Receptor for advanced glycation end product
AMPK: AMP-activated protein kinase
APC: Antigen-presenting cell
DC: Dendritic cells
IL: Interleukin
HMGB1: High mobility group box 1
CAFs: Cancer-associated fibroblasts
ROS: Reactive oxygen species
MSCs: Mesenchymal stem cells
ULK1: Unc51-like kinase 1
TNF-α: Tumor necrosis factor-α
NF-κB: Nuclear factor kappa B
MEC: Mammary epithelial cell
PARP: Poly-ADP-ribose polymerase
CQ: Chloroquine
HCQ: Hydroxychloroquine
